# Bone Damage Evolution Around Integrated Metal Screws Using X-Ray Tomography — *in situ* Pullout and Digital Volume Correlation

**DOI:** 10.3389/fbioe.2020.00934

**Published:** 2020-08-05

**Authors:** Sophie Le Cann, Erika Tudisco, Magnus Tägil, Stephen A. Hall, Hanna Isaksson

**Affiliations:** ^1^Department of Biomedical Engineering, Lund University, Lund, Sweden; ^2^Division of Geotechnical Engineering, Lund University, Lund, Sweden; ^3^Department of Orthopaedics, Clinical Sciences, Lund University, Lund, Sweden; ^4^Division of Solid Mechanics, Lund University, Lund, Sweden; ^5^Lund Institute for Advanced Neutron and X-ray Science, Lund, Sweden

**Keywords:** X-ray tomography, bone, metallic screw, *in situ* loading, Digital Volume Correlation

## Abstract

Better understanding of the local deformation of the bone network around metallic implants subjected to loading is of importance to assess the mechanical resistance of the bone-implant interface and limit implant failure. In this study, four titanium screws were osseointegrated into rat tibiae for 4 weeks and screw pullout was conducted *in situ* under x-ray microtomography, recording macroscopic mechanical behavior and full tomographies at multiple load steps before failure. Images were analyzed using Digital Volume Correlation (DVC) to access internal displacement and deformation fields during loading. A repeatable failure pattern was observed, where a ∼300–500 μm-thick envelope of bone detached from the trabecular structure. Fracture initiated close to the screw tip and propagated along the implant surface, at a distance of around 500 μm. Thus, the fracture pattern appeared to be influenced by the microstructure of the bone formed closely around the threads, which confirmed that the model is relevant for evaluating the effect of pharmacological treatments affecting local bone formation. Moreover, cracks at the tibial plateau were identified by DVC analysis of the tomographic images acquired during loading. Moderate strains were first distributed in the trabecular bone, which localized into higher strains regions with subsequent loading, revealing crack-formation not evident in the tomographic images. The *in situ* loading methodology followed by DVC is shown to be a powerful tool to study internal deformation and fracture behavior of the newly formed bone close to an implant when subjected to loading. A better understanding of the interface failure may help improve the outcome of surgical implants.

## Introduction

Metal implants are commonly used in surgery to e.g., stabilize bone fractures or replace joints or teeth. A joint prosthesis generally stabilizes biologically during the first year after implantation by integrating with the bone. Since the introduction of the concept of osseointegration (Bothe et al., 1940; Leventhal, 1951; Brånemark et al., 1969), much work has been done to increase knowledge about the bone-implant interface characteristics and behavior (Shah et al., 2019). However, poor bone ingrowth, linked for instance to increased implant micromotion, still compromises the long-term stability of the implant and leads to loosening and secondary surgery (Mathieu et al., 2014). Understanding the integration process and the strength of the newly formed bone-implant interface may help improve implant designs and surgical strategies in order to enhance prosthesis integration (Alenezi et al., 2018; Hanawa, 2019; Li et al., 2020).

The surrounding trabecular bone network has been shown to be important for the mechanical stability of implanted screws (Marquezan et al., 2014; Bernhardsson et al., 2015; Le Cann et al., 2019). Increased trabecular bone formation, by using, e.g., growth factors (Bernhardsson et al., 2015), or anti-resorptive drugs (Raina et al., 2019) improves the mechanical resistance of the bone-implant interface (Le Cann et al., 2019; Raina et al., 2019). The mechanical performance of the interface has until recently been assessed macroscopically during *in vitro* pullout tests, e.g., comparing bone volume fractions with obtained load-displacement data (Iijima et al., 2013; Shea et al., 2014; Raina et al., 2019). Such experimental data can be coupled with numerical models to investigate and predict the mechanical stability of implanted screws (Wirth et al., 2011; Chevalier et al., 2018). However, the trabecular bone structure is complex, and the understanding could be improved by investigating how the material deforms locally around an implant while being subjected to loading.

Mechanical loading under x-ray tomographic imaging, followed by image analysis such as Digital Volume Correlation (DVC), allows to access the internal deformation of a structured material subjected to load (Bay et al., 1999; Roberts et al., 2014; Grassi and Isaksson, 2015). Concurrent mechanical testing and imaging approaches have recently become widely accepted to track the load response of bone tissue and to study the evolution of damage and local failure (Dall’Ara et al., 2014; Gillard et al., 2014; Palanca et al., 2015; Jackman et al., 2016; Chen et al., 2017; Costa et al., 2017; Le Cann et al., 2017; Martelli and Perilli, 2018; Oliviero et al., 2018; Peña Fernández et al., 2018; Knowles et al., 2019). However, only sparse studies have applied the method to the bone-implant interface (Du et al., 2015; Sukjamsri et al., 2015; Joffre et al., 2017; Le Cann et al., 2017; Rapagna et al., 2019).

The choice of imaging modality is important to ensure sufficient image quality that is suitable for DVC. Synchrotron-based x-ray tomography currently provides the best signal to noise ratio and the fastest acquisition (Neldam et al., 2017). However, the high radiation dose may affect the mechanical properties of bone (Barth et al., 2011; Peña Fernández et al., 2018), and the fast imaging may result in strong artifacts around metallic implants (Le Cann et al., 2019). Lab-source micro-CT represents an alternative that is less detrimental in terms of radiation dose and thus preserving the mechanical properties (<1 kGy, Rawson et al., 2020) as well as potentially reducing the artifacts. However, lab micro-CT comes with the drawback of lower resolution and longer imaging times (Peyrin et al., 2014). Neutron tomography was recently presented as a promising alternative, allowing imaging of the implant-interface without any artifacts. However, neutron imaging comes with a cost of long imaging times and somewhat lower resolution (Isaksson et al., 2017; Le Cann et al., 2017).

This study investigates local damage in the bone around an implant during pullout, through concurrent x-ray tomography and mechanical loading followed by image analysis using DVC. Thereby, we aim (1) to assess the potential of DVC to characterize inner local displacements and deformations of newly formed bone around a screw during pullout and (2) to investigate the local failure patterns to validate the model’s usefulness to study possible treatments to improve bone regeneration using, e.g., delivery of growth factors.

## Materials and Methods

### Animal Model

A subset of four male Sprague Dawley rats that were part of a larger study is reported in this work. Overall, 40 rats (age 8 weeks, average weight 95 g ± 19 g, Charles River, Germany) were anesthetized with diazepam and pentobarbitalnatrium. Antibiotic prophylaxis was given as 12.5 mg dihydrostreptomycin and 10 mg procaine benzylpenicillin. A longitudinal incision over the anteromedial aspect of the right proximal tibial metaphysis was made under aseptic conditions. A hole with 1.5 mm in diameter was drilled transversely to the longitudinal axis of the bone to guide implantation of a titanium screw (Ø 2.6 mm, length 8 mm). Animals were treated with growth factors and anti-resorptive drugs to ensure early formation of substantial amounts of trabecular bone (Belfrage et al., 2012; Raina et al., 2016; Mathavan et al., 2018). Other subsets of these animals were previously used to develop the *in situ* methodology with neutron tomography (Le Cann et al., 2017) and synchrotron tomography (Le Cann et al., 2019). The previous studies highlighted that both biological treatments and surgical variability of the samples affected their mechanical response. To focus the current study on the fracture behavior of the bone-implant interface and the surrounding bone, we carefully isolated four samples with similar implant depth, distance from the tibial plateau and angulation of the screw. Each of these animals received 0.02 mL of BMP-7 (Osigraft, Stryker Biotech, Malmö, Sweden) in the form of a putty in the drilled hole before screw insertion. One animal was also given a systemic injection of Zoledronic Acid (0.1 mg/kg) 2 weeks after implantation (Zometa, Novartis, Apoteket AB, Sweden) to further increase the amount of trabecular bone by limiting premature bone resorption (Belfrage et al., 2012; Raina et al., 2016; Mathavan et al., 2018). The wound was closed, leaving the entire screw subcutaneous. After the operation, 4.5 μg buprenorphine of analgesic was given subcutaneously. All animal handling was approved by the regional animal research ethics committee (M25-13) and institutional guidelines for the care and treatment of experimental animals were followed. The rats had free access to water and food. The animals were sacrificed after 4 weeks of implant integration and the tibiae were carefully dissected with the screw in place and kept frozen (−20°C) until imaging.

### *In situ* Mechanical Pullout Test

**FIGURE 1:**
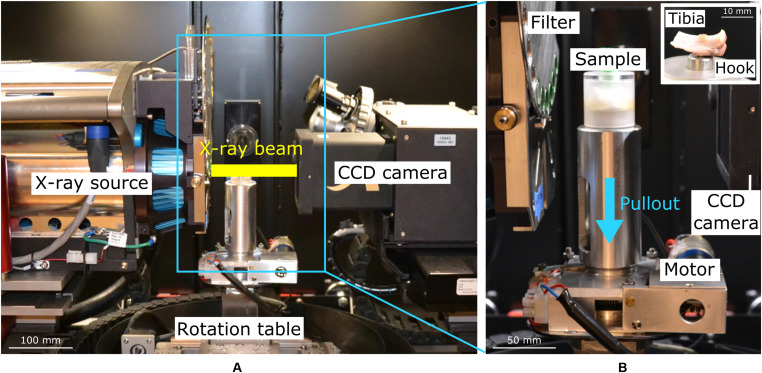
Overview of the x-ray μCT set-up **(A)** and zoom on the custom-made loading device **(B)** to pullout the screw *in situ*, with the sample wrapped in gauze inside the polycarbonate chamber. A view of the sample inside the chamber is presented in the insert. The blue arrow indicates the loading direction.

Screw pullout was conducted using a custom-made loading device placed in the x-ray tomograph (ZEISS XRM520 Versa) at the 4D Imaging Lab at Lund University (Figure 1A). The samples were wrapped in NaCl soaked gauze to limit dehydration during the test and placed in a closed cylindrical chamber with the screw head pointing downwards. The screw head was connected to a tensile rod through a hook and pulled out from the bone in displacement control (Figure 1B). The parts of the device that were in the x-ray path were made of polycarbonate to limit absorption. Force and displacement were monitored through a force-meter (U9C load cell, 500N, Hottinger Baldwin Messtechnik (HBM) GmbH) and a displacement sensor (cantilever-type with strain gauges, HBM) connected to the hook, and the data were recorded by a custom written LabVIEW program. All images for the tomographies were obtained with an energy of 80 kV (7 W, 87.5 μA) using a LE4 filter to remove the low energy x-rays and reduce the beam hardening artifacts (effective energy of the spectrum after filter is 77kV). A 20 × 20 × 20 mm^3^ field of view was used to include the whole tibia, leading to a 25 μm voxel size. 1601 radiographic projections over 360° were acquired with 1 s exposure time, resulting in a total scanning time of 70 min. The images were reconstructed with cone beam geometry using the software provided by the manufacturer (ZEISS).

A first, base-line, tomography was acquired after the initial loaded contact with the sample (preload of 5.9 ± 0.4 N). This is referred to as the “unloaded” scan. Immediately after the first scan, a displacement of 0.1 mm was applied at 0.1 mm/min, and after 400 s of relaxation, another tomographic acquisition was started. The procedure was repeated until a drop in the force-displacement curve was observed, followed by a final tomographic acquisition after failure.

### Analysis of Bone Formation and Screw Insertion

Peri-implant bone tissue was quantified in all samples using the base-line (first unloaded) scans and the image analysis software Fiji (Schindelin et al., 2012). First, images were filtered (Median filter radius 4 voxels) and the screw was aligned vertically using the plugin TransformJ (Meijering et al., 2001). Images were then visually binarized to remove the background and the screw using the same thresholds for all samples (IsoData algorithm, Fiji). A cylindrical region of interest (ROI) of 0.5 mm extending from the screw maximal diameter and spanning over the threads (2.2 mm length) was defined and bone volume fraction (bone volume/total volume, BV/TV) was quantified inside the ROIs using the plugin BoneJ (Doube et al., 2010).

Screw insertion was evaluated by measuring the distance and the tilt between the screw and the tibial plateau (Le Cann et al., 2019) on 2D radiographs (pixel size 150 μm, GE Healthcare discovery x-ray machine, CT, United States). The screw-tibial plateau distance was defined as the orthogonal projection of the middle of the screw threads onto the tibial plateau line, between the two intercondylar eminences. The screw tilt corresponded to the angle between the long axis of the screw and the tibial plateau line. Pearson correlations were used to investigate relationships between screw insertion and mechanical parameters.

### Digital Volume Correlation (DVC)

To assess internal local damage during loading, the 3D images were analyzed using DVC with the python-based in-house DVC code TomoWarp2 (Le Cann et al., 2017; Tudisco et al., 2017). This local-DVC approach tracks image intensities in subsets between the first reference image and a consecutive deformed image to access 3D displacement and deformation fields (Bay et al., 1999; Roberts et al., 2014). For a more complete description, please see Hall et al. (2010) and Tudisco et al. (2017).

A regular, cubic grid with node spacing of 4 voxels (representing the grid of analysis points) and cubic subsets of 17 voxels per side (around each node of the grid) were used for the analysis, leading to a voxel size of 100 μm in the DVC maps. Based on two gray value thresholds, the background and the screw were removed from the analysis by setting the corresponding voxels values to NaNs, i.e., these voxels were not included in the calculation of the correlation coefficient to avoid any edge-effect. Repeated over the entire grid points, 3D displacement fields were extracted between two loading steps and smoothed by a median filter (radius 1 voxel) before calculating strains using a continuum mechanics approach. The Green-Lagrange strain tensor field was obtained using linear shape functions on 8-node isoparametric hexahedron finite elements whose nodes correspond to the grid points. Volumetric strain is then calculated as det(**F**)-1, where **F** is the deformation gradient, and the maximum shear strain according to Eq. 1.

(1) $$\frac{1}{3}\sqrt{2{\left(\varepsilon_{xx}-\varepsilon_{yy}\right)}^{2}+2{\left(\varepsilon_{xx}-\varepsilon_{zz}\right)}^{2}+2{\left(\varepsilon_{yy}-\varepsilon_{zz}\right)}^{2}+12\varepsilon_{yx}^{2}+12\varepsilon_{zx}^{2}+12\varepsilon_{zy}^{2}}$$

Accuracy and precision of the DVC measurements were determined from two repeated unloaded scans of one sample. The same DVC approach was applied and the errors were quantified using Matlab, calculating the mean (accuracy of the procedure) and standard deviation (precision) of each displacement component (X, Y, and Z-displacements) and of all absolute values of the six strain components.

## Results

### Baseline Imaging

Analysis of the unloaded baseline scans revealed an average bone volume fraction around the screw of 32 ± 0.5% (range 28–33), screw-tibial plateau distance of 7.2 ± 1.2 mm (5.7–8.5) and screw tilt of 9.9 ± 7.6° (5.2–21).

### *In situ* Loading and Macro-Mechanics

Between four and eight scans were acquired per sample, resulting in a total testing time of 7–11 h. The mechanical behavior was similar among the samples, with average stiffness of 161 ± 68 N.mm^–1^ (65–215) and maximum force of 38 ± 11 N (26–51). No correlation was found between screw tilt and the measured macroscopic mechanical properties. However, the displacement at maximum force correlated to the screw-tibial plateau distance (*R*^2^ = 0.98, *P* = 0.012), with a higher displacement at maximum force recorded when the screw was closer to the tibial plateau.

### Screw Pullout: Failure Away From the Screw-Bone Interface

**FIGURE 2:**
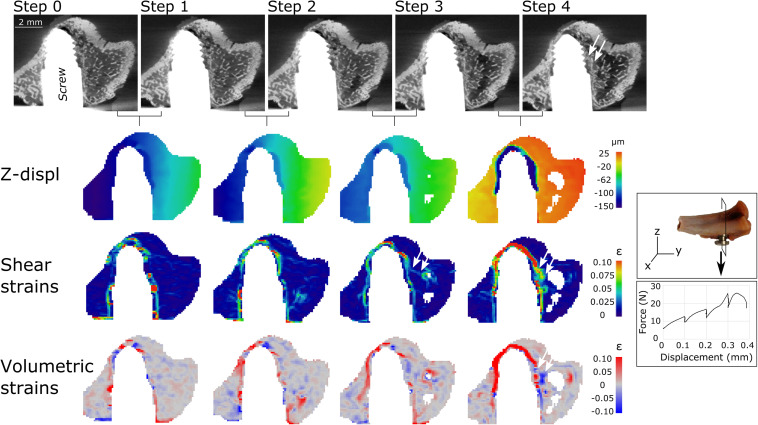
Tomography scan cuts (top row) after each loading step for sample S2. Cutting direction of the scans is presented in the insert to the right. Three lower rows present the corresponding DVC results between loading steps, with vertical displacements (Z-displ), shear and volumetric strains. Bone detachment close to the threads can be observed in the last scan (step 4). High strains were already detected step 2–3 (before failure) where the crack will later develop (arrows).

**FIGURE 3:**
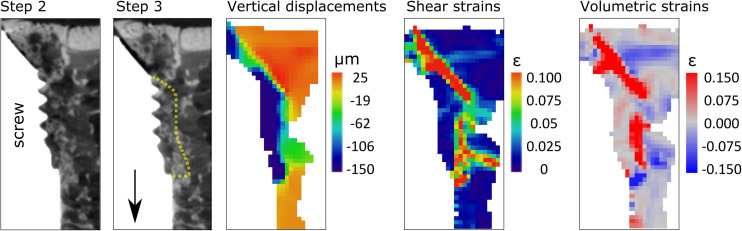
Zoom in on two last steps of Sample S4, to illustrate the detachment of the bone close to the threads from the trabecular network. From left to right, tomographic slices with yellow dotted line highlighting the bone detaching, and DVC results for the same position.

A similar mechanical behavior was observed in all samples, and the results are illustrated based on two samples. Consistently, trabecular bone attached to the screw threads appeared to follow the screw pullout direction, while the rest of the sample remained steady (Figures 2, 3 and Supplementary Data Sheet 1, File 1). This fracture pattern with detachment of trabecular bone was visually observed on all tomographic images after failure, but it was already detected in the DVC results one to two steps before failure in the form of propagation of the crack(s) inside the trabecular network (Figure 2 and Supplementary Data Sheet 1, File 1). For instance, compressive strains (negative volumetric strains) were detected in regions where bone detached from the trabecular network and was crushed (blue, lowest row Figures 2, 3). Visually in the tomographies, the flat surfaces of the implant (screw tip and cylindrical part close to the hook) appear to detach and slide, as highlighted by the DVC analysis with dilatational strains (positive volumetric strains) at crack opening (red, lowest row Figures 2, 3).

The DVC analysis showed that the displacements in the loading direction were substantially higher close to the interface at one-two steps before maximum load, compared to the rest of the sample (Figures 2, 3). This region of higher displacements formed an envelope around the threaded region of the screw, which was about 300- to 500 μm-thick with displacement magnitude of about 100 μm (4 voxels) between two loading steps, which corresponded well with the displacement step applied to the screw.

**FIGURE 4:**
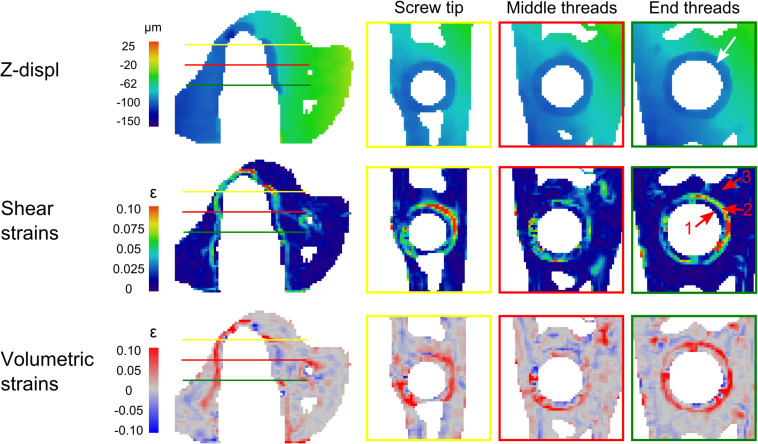
The left column represents the same cutting direction as Figure 2. Right side shows 3 horizontal cuts of the threaded region: close to the screw tip (yellow line), the middle (red) and close to the end of the screw threads (green). Note the envelope of vertical displacement (∼4 voxels) around the screw (white arrow) inducing a specific strain pattern with a strain-free regions (1, red arrow), followed by high strains region (2, red arrow), and again a low strain region (3, red arrow).

The envelope of high displacement induced a shell of high strains around the screw threads (lower rows Figures 2, 4). Three main regions were observed: a strain-free region close to the threads (labeled 1 in Figure 4), followed by a high strain region at a distance of 300–500 μm (3–5 DVC voxels) from the interface where the high displacement region ends (labeled 2), and finally a low strain region continuing into the trabecular structure (labeled 3).

### Crack Development at the Tibial Plateau

**FIGURE 5:**
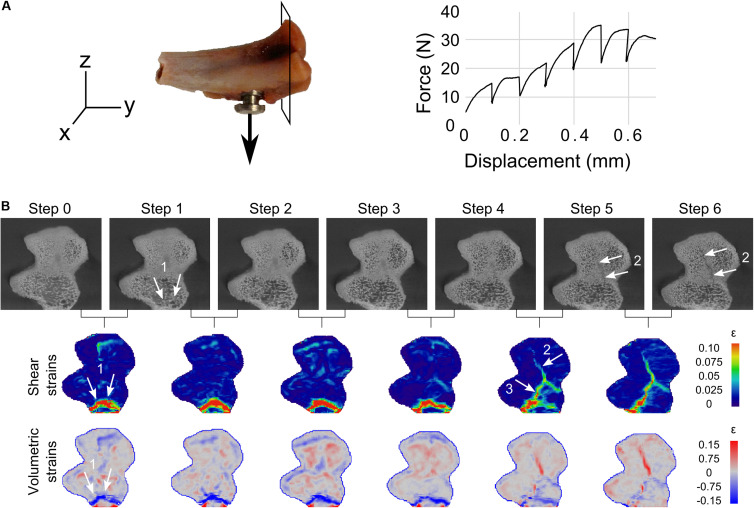
**(A)** Cutting direction for the following results and loading curve of sample S1. **(B)** From top row to bottom row, tomographic scans cuts at the tibial plateau region, and corresponding DVC shear and volumetric strains for all loading steps. Crushing of bone close to the support (arrows 1) can be seen in the scans (see also Supplementary Data Sheet 1, Files 2, 3). Crack opening (2) is also seen in scans in relatively dense bone. However, the connection (3) between 1 and 2 was not clearly observed in the tomographies (see Supplementary Data Sheet 1, File 3). Note the strains spread in the bone structure at early loading stages, gathering where the cracks are to open, and finally relaxing in the bone while the cracks were opening (from step 4–5).

In addition to the deformation around the screw, cracks were also seen to develop at the tibial plateau in two samples (sample S1 and sample S3) during the pullout. The results are presented for sample S1 (Figure 5 and Supplementary Data Sheet 1, Files 2, 3). During the early loading (steps 0–4), bone at the tibial plateau was compressed and crushed close to the support (see Figure 5B, label 1 and Supplementary Data Sheet 1, File 3) while moderate strains were spread out inside the trabecular bone. These strains localized (step 4–5) and a crack appeared (label 2, step 5). Moreover, a third crack (labeled 3), linking cracks 1 and 2, was revealed by the DVC analysis that could not be clearly seen in the tomographies (Supplementary Data Sheet 1, File 3). During the final loading steps, the cracks continued to open and propagate inside the trabecular structure toward the implant (see Supplementary Data Sheet 1, File 2), while the strains relaxed in the remaining bone tissue.

The errors from the DVC calculations assessed on a repeated unloaded scan revealed accuracy and precision below 6.2 μm and 3.6 μm for displacements (only maximum values reported), and accuracy and precision of 4900 and 7400 με for strains.

## Discussion

This study highlights the benefits of investigating local bone damage close to an osseointegrated implant with concurrent mechanical loading and tomographic x-ray imaging, followed by image analysis with DVC. We found a typical failure pattern through an envelope of bone that detached from the trabecular structure, suggesting that fracture was initiated close to the screw tip and propagated at some distance away from the implant surface.

This study was part of a larger framework that investigated the use of growth factors to promote new bone formation around implants. Our aim was not to investigate the impact of the growth factors, as we have already shown that BMP alone or in combination with Zoledronic Acid promotes trabecular bone formation in a variety of small animal models (Bosemark et al., 2013; Raina et al., 2016, 2019; Mathavan et al., 2018). This animal model has been designed to particularly test the trabecular bone’s resistance to pullout. The sliding observed between the bone and the implant in the implant that was not threaded (cylindrical portion) confirms that the cortical bone had a limited influence in this model (Le Cann et al., 2019).

Previous work has shown that a combination of various parameters effect the mechanical behavior of the screw-bone construct, including screw insertion position, angle and depth, as well as peri-implant bone content (Varghese et al., 2017; Le Cann et al., 2019). For this study, we focus on bone-implant failure and limit other sources of variability by isolating, from a larger set, four samples that presented similar screw-tibial plateau distance, peri-implant bone content and macro mechanical response during pullout (stiffness and maximum force). Additionally, the BV/TV values were consistent with previous analyses on the same screw-ingrowth model when imaged with higher resolution (Le Cann et al., 2019).

The errors of the DVC procedure were estimated from a repeated scan, and despite being higher than usual due to metal artifacts, they still remained within the range of values presented in literature (e.g., Dall’Ara et al., 2017; Tozzi et al., 2017). It has to be noted that the strain values are not of interest here but are used as apparent strains to localize and investigate the fracture behavior, as we did not differentiate continuous vs. discontinuous (broken) regions.

All four samples experienced screw pullout, as observed in the tomographic images after failure and further revealed by DVC. The DVC images revealed an envelope of vertical displacements (pullout direction) about 1–2 steps before failure that started to form around the threads. The magnitude was approximately 100 μm (i.e., 4 voxels), which corresponded well with the loading step size. This suggests that the failure of the interface starts at the screw tip and propagates relatively far from the surface (∼300–500 μm). Bone tissue close to the threads stayed attached to the implant without being deformed, but detached from the trabecular network (Figure 3) where a shell of high strains is observed. This is consistent with observations made directly from the tomographic images and can be explained by the rather smooth screw threads. To our knowledge, only one other study experimentally investigated screw pullout using *in situ* loading (Joffre et al., 2017). They observed strains initiating in close proximity of the implant (<500 μm), and decreasing radially inside the bone (up to 2 mm), which they attributed to bending of the trabeculae. The current study differs when it comes to the screw design (approximately 1 mm long threads as opposed to 200 μm in our screw model) and the animal model (rabbit vs. rat in our study). Most importantly, the screws in Joffre et al. (2017) were not osseointegrated, which most likely affects the failure mechanisms. Our smoother thread design combined with denser trabecular bone lead to a concentration of strains around the screw, with a quick decrease in strain intensity going away from the threads inside the trabecular structure. As the failure pattern was observed close to the screw, it appears to be highly dependent on the bone microstructure formed around the threads, and thus represents an interesting model to investigate the effect of local differences in bone close to the implant surface, which can be highly affected by local drug treatment (Raina et al., 2019). Combined with the *in situ* loading methodology, this model is valuable to understand how screw-bone interfaces are loaded in order to improve designs of screws and to investigate the effect of treatments on trabecular bone.

The image quality close to the implant was reduced because of artifacts due to the high difference in absorption between titanium and bone, as is often the case with x-rays based methods (Barrett and Keat, 2004). Such artifacts usually disturb the images up to 60 μm from the metal surface (Li et al., 2014), and it has been recommended to examine the interface within 200 μm with caution (Vandeweghe et al., 2013). The voxel size of the tomographic scans of 25 μm lead to a relatively high image analysis resolution (DVC voxel size of 100 μm as a consequence of the node spacing of 4 voxels), so the artifacts did not seemingly disturb the analysis too much, as the main strain patterns were observed 300–500 μm away from the implant. Nonetheless, such artifacts could have hindered the analysis of possible bone damage initiated directly at the implant surface, around the screw threads and especially at the tip where gap opening is observed. Artifact-free images of higher resolution would be needed to more carefully investigate the failure pattern of this envelope of peri-implant newly formed bone.

In addition to the deformation around the implant, two samples also cracked at the tibial plateau far away from the interface. This was a result of the experimental design, where forces were channeled through the cortical shell during pullout, as previously observed (Le Cann et al., 2017). Tibial fractures were successfully detected by the DVC technique in the form of high apparent strains prior to crack formation (Figure 5 and Supplementary Data Sheet 1, Files 2, 3), which were not all visible in the tomographic images. Moreover, DVC also provided insight onto the formation of those cracks showing strain concentration at the crack zone prior to crack appearance (Figure 5 and Supplementary Data Sheet 1, File 3, before step 4). This confirms the usefulness of the DVC technique to track crack formation and evolution in bone during loading.

The most important limitations of the study are linked to the use of the DVC technique, which was found more efficient in regions far from the screw where metal artifacts were minor. Moreover, dehydration from long imaging time occurred in trabecular bone, as observed in Figure 2, which led to a loss of DVC points. Future efforts will be dedicated to acquiring artifact-free images, for instance using neutron tomography (Le Cann et al., 2017), or less absorbant implants (PEEK, ceramics) as well as developing image analysis filters or techniques to correct for artifacts, mostly implemented for clinical-CT so far (Katsura et al., 2018).

## Conclusion

*In situ* loading combined with Digital Volume Correlation is becoming a powerful tool to investigate bone damage close to an implant. In this study, DVC analysis was applied to a series of x-ray tomographic images obtained during mechanical pullout of an osseointegrated implant. The methodology revealed a consistent failure pattern, in the close trabecular bone structure around the screw threads, at an approximate distance of 300–500 μm, suggesting that this model is well-suited to evaluate the effect of drugs or other treatment to increase bone formation. Further investigations will be needed at higher resolution to investigate and resolve the finer processes at the direct interface and especially the crack initiation, which our data points to that it happens at the screw tip.

## Data Availability Statement

All data generated or analyzed during this study are included in this published article and its Supplementary Information Files. Additional datasets are available from the corresponding author on reasonable request.

## Ethics Statement

The animal study was reviewed and approved by the Ethics Committee affiliated with the Swedish Board of agriculture (Jordbruksverket, Jönköping, Sweden), under the animal ethics permission number M25-13.

## Author Contributions

SL, ET, SH, and HI designed the study, planned, and carried out the *in situ* imaging experiment. MT conducted the animal experiments. SL analyzed the data and wrote the draft of the manuscript together with HI. All authors contributed to the article and approved the submitted version.

## Conflict of Interest

The authors declare that the research was conducted in the absence of any commercial or financial relationships that could be construed as a potential conflict of interest.
