# Supplementary material for: Bone Damage Evolution Around Integrated Metal Screws Using X-Ray Tomography — in situ Pullout and Digital Volume Correlation
Source: Front Bioeng Biotechnol. 2020 Aug 5;8:934. doi: 10.3389/fbioe.2020.00934 (PMC7419699; doi:10.3389/fbioe.2020.00934)
Supplement: Supplementary file 1 [file Data_Sheet_1.PDF]

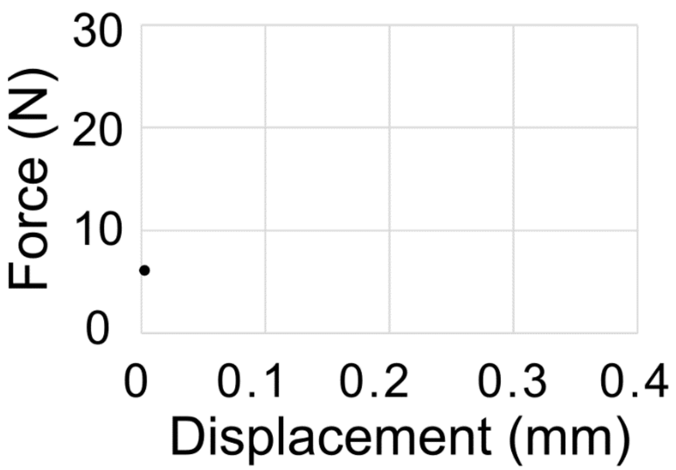

Scan - step 0

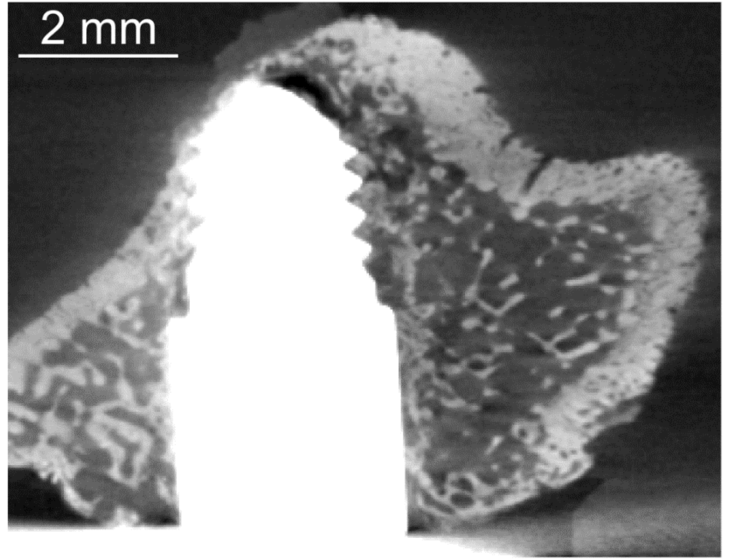

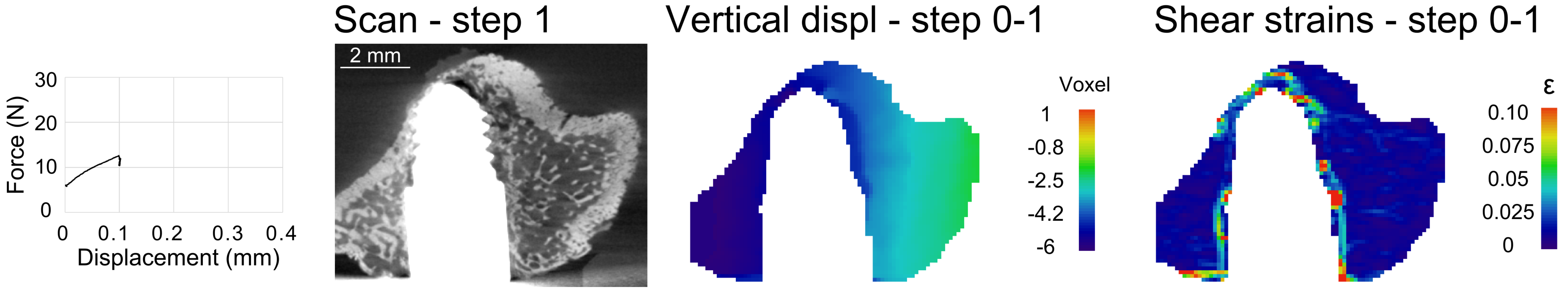

Force (N)
30
20
10
0
0
0.1
0.2
0.3
0.4
Displacement (mm)
Scan - step 1
2 mm
Vertical displ - step 0-1
Voxel
1
-0.8
-2.5
-4.2
-6
Shear strains - step 0-1
ε
0.10
0.075
0.05
0.025
0

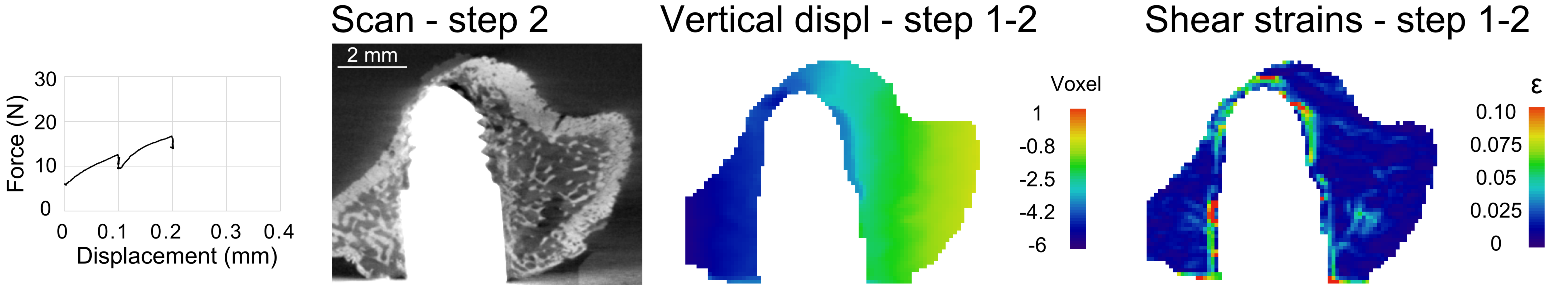

Scan - step 2
Vertical displ - step 1-2
Shear strains - step 1-2
30
20
10
0
Force (N)
0
0.1
0.2
0.3
0.4
Displacement (mm)
2 mm
Voxel
1
-0.8
-2.5
-4.2
-6
ε
0.10
0.075
0.05
0.025
0

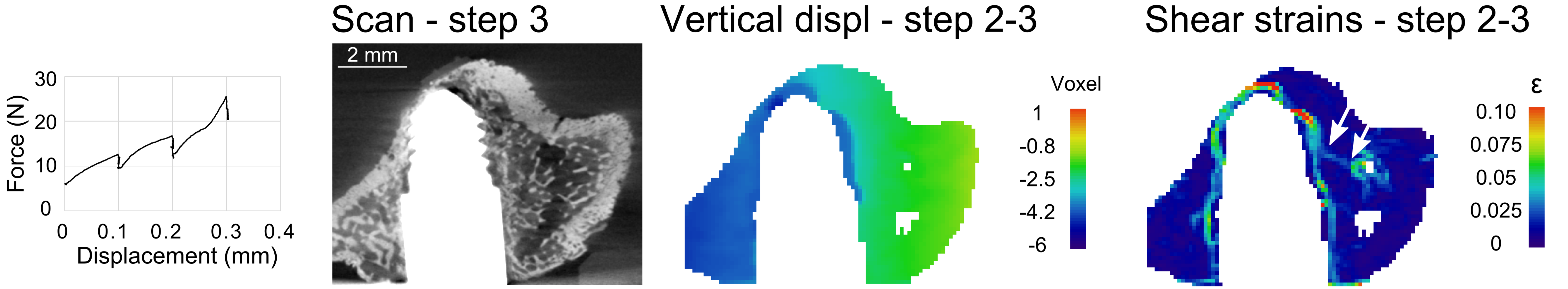

Scan - step 3
Vertical displ - step 2-3
Shear strains - step 2-3
30
20
10
0
Force (N)
0
0.1
0.2
0.3
0.4
Displacement (mm)
2 mm
Voxel
1
-0.8
-2.5
-4.2
-6
ε
0.10
0.075
0.05
0.025
0

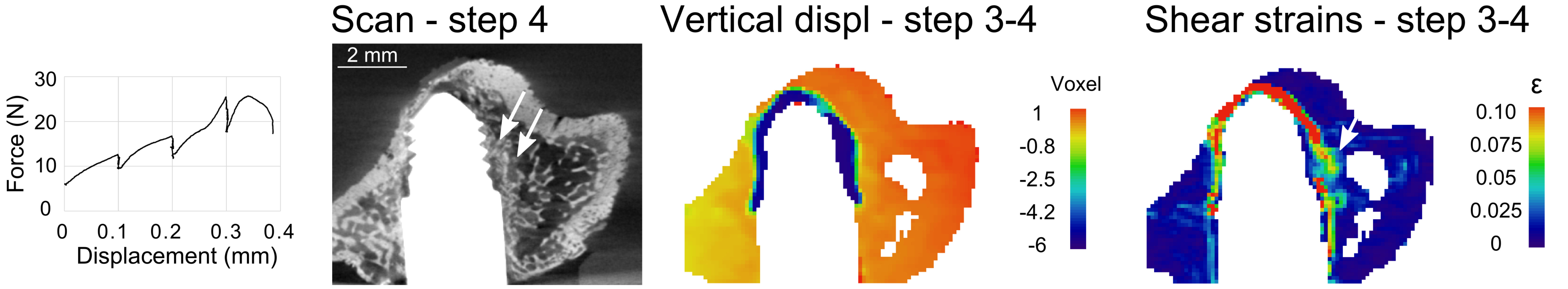

Force (N)
30
20
10
0
0
0.1
0.2
0.3
0.4
Displacement (mm)
Scan - step 4
2 mm
Vertical displ - step 3-4
Voxel
1
-0.8
-2.5
-4.2
-6
Shear strains - step 3-4
ε
0.10
0.075
0.05
0.025
0
